# Sentinel lymph node procedure in patients with recurrent vulvar squamous cell carcinoma: a proposed protocol for a multicentre observational study

**DOI:** 10.1186/s12885-022-09543-y

**Published:** 2022-04-23

**Authors:** Helena C. van Doorn, Maaike H. M. Oonk, Guus Fons, Katja N. Gaarenstroom, Joanne de Hullu, Joost van Rosmalen, Heleen J. van Beekhuizen

**Affiliations:** 1 Department of Gynaecologic Oncology, Erasmus MC Cancer Institute, University Medical Center Rotterdam, P.O. Box 2040, 3000 CA Rotterdam, The Netherlands; 2 Department of Obstetrics and Gynaecology, University Medical Center Groningen, University of Groningen, Groningen, The Netherlands; 3 Department of Obstetrics and Gynaecology, Amsterdam UMC, Amsterdam, The Netherlands; 4 Department of Obstetrics and Gynaecology, Leids University Medical Center, Leiden, The Netherlands; 5 Department of Obstetrics and Gynaecology, Radboud University Medical Center, Nijmegen, The Netherlands; 6 Department of Biostatistics, Erasmus MC, University Medical Center Rotterdam, P.O. Box 2040, 3000 CA Rotterdam, The Netherlands; 7 Department of Epidemiology, Erasmus MC University Medical Center Rotterdam, Rotterdam, The Netherlands

**Keywords:** Vulvar cancer, Recurrence, Sentinel lymph node

## Abstract

**Background:**

Standard groin treatment in recurrent vulvar cancer consists of uni- or bilateral inguinofemoral lymphadenectomy (IFL), whereas in the primary setting women with selected unifocal tumours will undergo a sentinel lymph node (SLN) procedure. The SLN procedure results in fewer short and long-term sequelae compared to IFL, but some concerns must first be considered. Lymph drainage of the vulvar region can be affected by a previous surgery, which might reduce the number of detectable SLN nodes (feasibility) but increase the chance of encountering aberrant lymph drainage patterns such as bilateral SLNs in lateral tumours or SLNs at unexpected locations. Therefore, the SLN procedure potentially carries a higher risk of groin recurrence if a tumour positive node is not retrieved, but may also improve outcomes for women with aberrant drainage patterns. Since the relative benefits and drawbacks of the SLN procedure are still unclear we will investigate the safety of the SLN procedure in women with a first recurrent vulvar cancer. In a simultaneously started registration study we prospectively gather information on women with a first recurrence of vulvar cancer ineligible for the SLN procedure.

**Method:**

In this prospective multicentre observational study all women with a first recurrence of vulvar cancer will be asked to consent to the collection of information on their diagnostics, treatment and outcome, and to complete quality of life and lymph oedema questionnaires. Women with unifocal tumours smaller than 4 cm and unsuspicious groin nodes will be offered the SLN procedure, with follow-up every 3 months together with imaging at 6 and 12 months when the SLN is tumour negative. The primary outcome is groin recurrence within 2 years of initial surgery. A total of 150 women with negative SLNs will be required to demonstrate safety, a stopping rule will apply and an extensive statistical analysis has been designed.

**Discussion:**

Should the SLN procedure prove feasible and safe in recurrent vulvar cancer, it will be available for implementation in clinics worldwide. The inclusion of women ineligible for the SLN procedure in the current prospective study will help to bridge knowledge gaps and define future research questions.

**Trial registration:**

Medical Ethical Committee approval number NL70149.078.19 (trial protocol version 2.0, date March 2nd, 2020). Affiliation: Erasmus Medical Centre. Dutch trial register NL8467. Date of registration 19.03.2020.

## Background

The treatment of primary vulvar cancer has shifted over the last decades. The current standard treatment for unifocal squamous cell carcinoma of the vulva (V-SCC) less than 4 cm in diameter and without suspicious inguinofemoral lymph nodes at imaging consists of wide local excision and a sentinel lymph node (SLN) procedure of the inguinofemoral lymph nodes [1–4]. The advantages of the SLN procedure over an inguinofemoral lymphadenectomy (IFL) are obvious: short and long-term sequelae such as wound healing problems, lymph cyst formation, recurrent erysipelas and lymph oedema are much less common following an SLN procedure [3]. Groin recurrences after negative SLN procedures in primary V-SCC patients were reported in 2–3% of the patients [3, 5]. The prognosis of a groin recurrence after a negative SLN procedure is unclear, but groin recurrence after IFL has a very high mortality rate [6–9]. Therefore, very strict criteria with respect to tumour characteristics, preoperative and pathological assessment and surgical technique should be met to guarantee the safety of the SLN procedure and to minimize the number of false negative results and the risk of groin recurrence.

Reliable prospective follow-up data after a first episode of vulvar SCC are not available, but local recurrences of SCC of the vulva are reported in 20–46% of cases [10, 11]. Many of these recurrences might be second primary tumours on a background of lichen sclerosis and differentiated vulvar intraepithelial neoplasia or a high-grade squamous cell intraepithelial lesion of the vulva rather than a real recurrence [12, 13]. In patients with recurrent vulvar SCC, IFL is considered the standard treatment for patients who have not previously undergone a (bilateral) IFL [14, 15]. In a previous retrospective study that included 292 women with a macro-invasive local recurrence, the 16% risk of groin metastases was related to depth of invasion and tumour diameter of the recurrent tumour [16]. In the same study we found that, in a deviation from the guideline, groin surgery had not been performed in a considerable number of patients [10, 16].

In an earlier multicentre retrospective study we showed that an SLN procedure seems feasible in recurrent V-SCC, as the SLN procedure could be performed as planned in 77% of 27 patients and in 84% of the 44 groins [17]. In two groins, SLNs were found at unexpected locations beyond the borders of normal IFL, and four lateral tumours showed bilateral SLNs. Data on safety are lacking but so far, none of the patients with a negative SLN procedure in the study has suffered groin or distant recurrences after a median follow-up of 27 months (range 2–96 months). We concluded that the SLN procedure might be helpful in the visualization of lymph drainage and guides the gynaecologic oncologist in the removal of at risk lymph nodes.

Since many vulvar cancer patients are elderly and frail, an alternative treatment to IFL may be justified to avoid or reduce long-term morbidity in this particular group of patients. Currently we have little understanding of the decisions that underlie treatment choices in women with recurrent vulvar cancer, i.e., it is not clear whether these women would choose for limited surgery such as SLN when surgery that is more extensive is the standard of care, or why women would prefer to undergo only a limited local surgical procedure, without groin treatment.

All women with a first vulvar cancer recurrence will be included in an observational study, notwithstanding previous and planned treatment, with the aim of achieving a better appreciation of the magnitude of the clinical challenges and treatment outcomes, with the eventual aim of optimizing information for patients and formulating further research goals.

Women with a first recurrent vulvar SCC smaller than 4 cm will be offered an SLN procedure as an alternative to a complete IFL. Our overall aim is to investigate the feasibility and safety of the SLN procedure in patients with a first recurrence of vulvar SCC.

## Methods/design

### Setting and study population

**Fig. 1:**
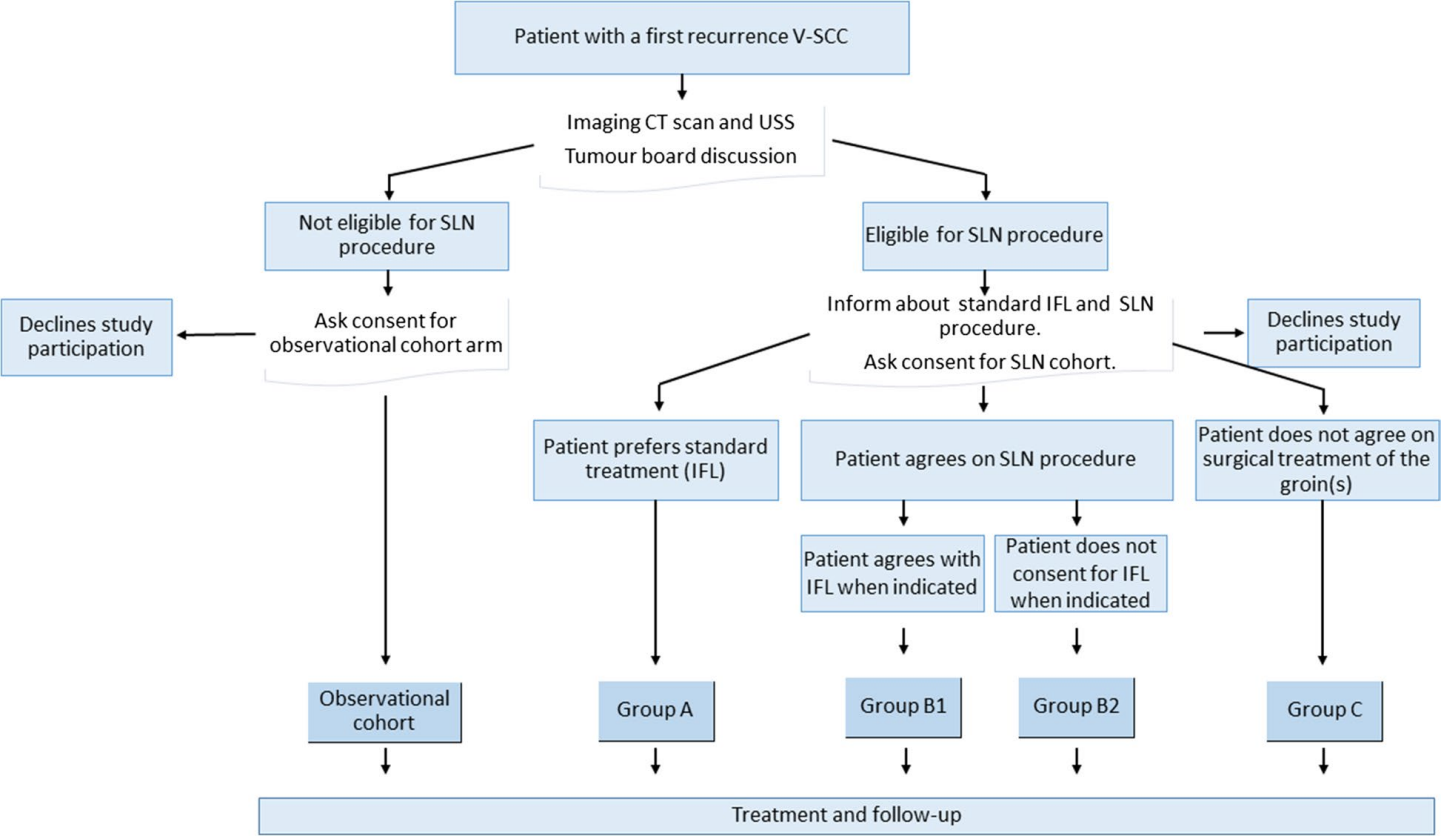
Flowchart of the study design. Legend: SLN: Sentinel Lymph node, V-SCC; vulvar squamous cell cancer. USS; ultrasound scan of the groins, IFL Inguinofemoral lymphadenectomy

This prospective multicentre observational study uses the acronym V2SLN and will commence in five university hospitals in the Netherlands that all have considerable experience with the necessary techniques (see authors). In participating centres, all patients with a first vulvar cancer recurrence will be registered and asked to take part in the observational study. Standard imaging (i.e., chest, abdominal and groin CT scanning) will be performed. The patient will be discussed at the local tumour board and a treatment recommendation will be defined. Women with a unifocal tumour smaller than 4 cm, without distant or groin metastases, and fit for surgery will be candidates for substituting the SLN for the IFL procedure (Fig. 1).

Patients will be informed using oral and written information about both the standard treatment (i.e. IFL) and the SLN procedure as part of the study. Patients will be advised of the possibility that the SLN may not be identified by injection or at surgery and in this case the standard treatment should be uni- or bilateral IFL. However, in our retrospective study, not all patients gave consent for groin treatment and some seemed particularly averse to IFL [17].

We will compare the following three groups of women with recurrent vulvar cancer in which an SLN procedure is feasible:

1. Women who choose to undergo IFL despite the alternatives.
2. Women who prefer SLN. In a number of cases the SLN procedure will not be feasible and these patients will be asked to decide, prior to surgery, whether in that case an IFL(s) should be performed.
3. Women who prefer no treatment in the groin regions.

Patients in all three groups will be asked to give consent for the collection of information regarding treatments and outcomes and to complete the questionnaires.

### Inclusion criteria

#### Eligibility for the observational study

First local recurrent SCC of the vulva, regardless previous treatment of the vulva or groin.

- Ability to understand and read Dutch.
- Able to understand the study and give informed consent.
- No age limit specified, but by nature of the tumour, all will be > 18 years.
- Patients should be mentally, physically and geographically able to undergo follow-up.

#### Eligibility to undergo the SLN procedure

- Previous treatment with wide local excision or (partial) vulvectomy.
- The tumour measures 4 cm or less and does not encroach on the urethra, vagina or anus.
- Localisation of the tumour is such that perilesional injection of the tracers at three or four sites is possible.
- Clinically-negative inguinofemoral lymph nodes and preoperative imaging does not show enlarged (> 10 mm sort axis) or suspicious nodes.
- Fit for surgery.

### Exclusion criteria for the study arm

A potential subject who meets any of the following criteria should not undergo the SLN procedure in the study arm.

- Multifocal recurrent disease of the vulva.
- Previous surgery of the vulva was not radical (margin < 1 mm) and additional treatment (second surgery or radiotherapy) was not performed.
- A history of bilateral IFL and radiotherapy to the groins.
- A lateral tumour and history of ipsilateral IFL and ipsilateral radiotherapy.
- Synchronous, non-curable second malignancy.

### Primary outcome

The main study outcome is the number of women who develop a groin recurrence within 24 months after a technically successful SLN procedure in which the SLN was free from tumour in recurrent vulvar cancer.

### Secondary outcomes

- Feasibility of the SLN procedure in a first recurrent V-SCC
- Finding of unexpected SLN’s
- Number of tumour-positive SLN’s and groins
- Outcome of quality of life questionnaires
- Patient’s preferences
- Surgical complications
- Sonography of groins at follow-up
- Percentage of groin recurrence after (standard) IFL procedure

### Intervention SLN procedure

Prior to the procedure the patient and her treatment team discuss and document what the surgery is aiming for and which surgery should take place in cases where the SLN procedure is not feasible. Women who undergo an SLN procedure receive 3 to 4 intradermal injections circumferentially around the tumour of 99mTc-labeled Nano colloid with a particle size < 80 nm. In accordance with the local protocol either patent blue or a fluorescent technique is used to visualize the SLN(s). The surgical sequence groin-vulva, or vice versa, is decided upon during surgery.

Pathological ultra-staging will be performed on SLNs and the reporting includes number of SLNs, number of lymph nodes in lymphadenectomy specimen, number of nodes with metastatic involvement, size of lymph node metastasis and presence or absence of extra nodal tumour growth.

### Data collection

Coded data will be stored both on paper and in an electronic database. Patient characteristics will be stored in an electronic database. Collected data will be stored in a digital case report form (CRF) and the raw data will only be available to the principal and coordinating investigator. A CRF will be completed preoperatively, postoperatively, and at 3, 6, 9, 12, 15, 18, 21 and 24 months postoperatively. Prior to surgery and at 6, and 12 months postoperatively, all participants will complete a quality of life questionnaire (EQ-5D-5L, adjusted GCLQ), and women eligible for the SLN procedure will complete a Decisional Conflict Scale and Decision Regret Scale.

The following data will be recorded:

- *Preoperatively*: Patient characteristics, tumour characteristics (first and current episode), outcome of preoperative imaging, signed PIF and treatment decision form.
- *(Post)operatively*: operative parameters, outcome of surgery, postoperative (re)hospitalization, complications (wound healing), histology outcome, and proposed and executed adjuvant treatment.
- *Follow- up:* local and regional (disease) status, complications (erysipelas, wound problems, symptomatic lymph oedema) at 6 and 12 months. For women with a tumour negative SLN procedure ultrasonography of groins at 6 and 12 months.

The datasets used and/or analysed during the current study will be available from the corresponding author on reasonable request after publication of the study results.

### Statistical considerations

**Fig. 2:**
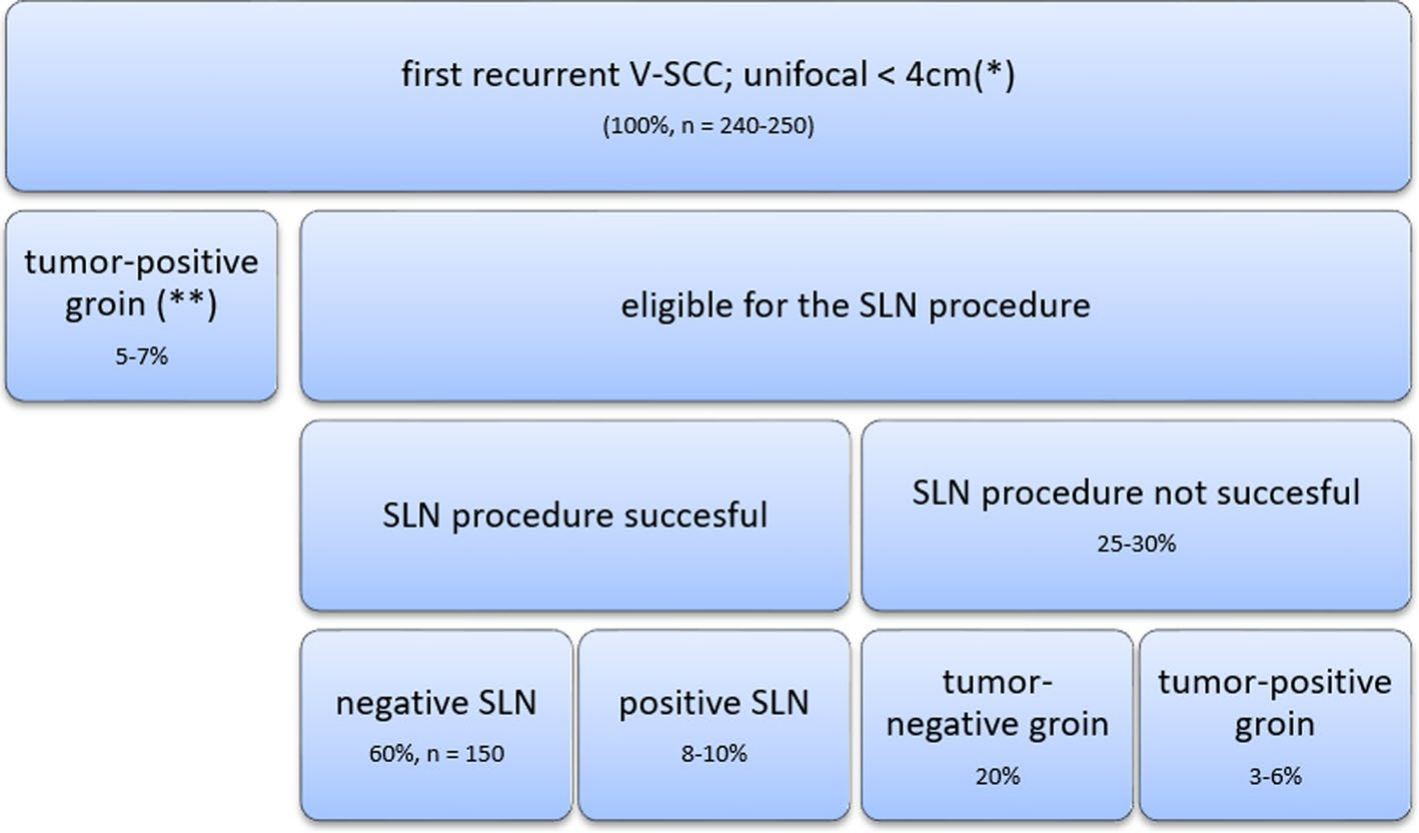
Estimated guess of sample size to include 150 cases with a tumour negative SLN. Legend: SLN: Sentinel Lymph node, V-SCC; vulvar squamous cell cancer. * see text for all in- and exclusion criteria, **inclusion and exclusion criteria applicable to local tumour, but strong suspicion or evidence of tumour positive lymph nodes (clinical, radiological or proven with fine needle aspiration or biopsy)

**Fig. 3:**
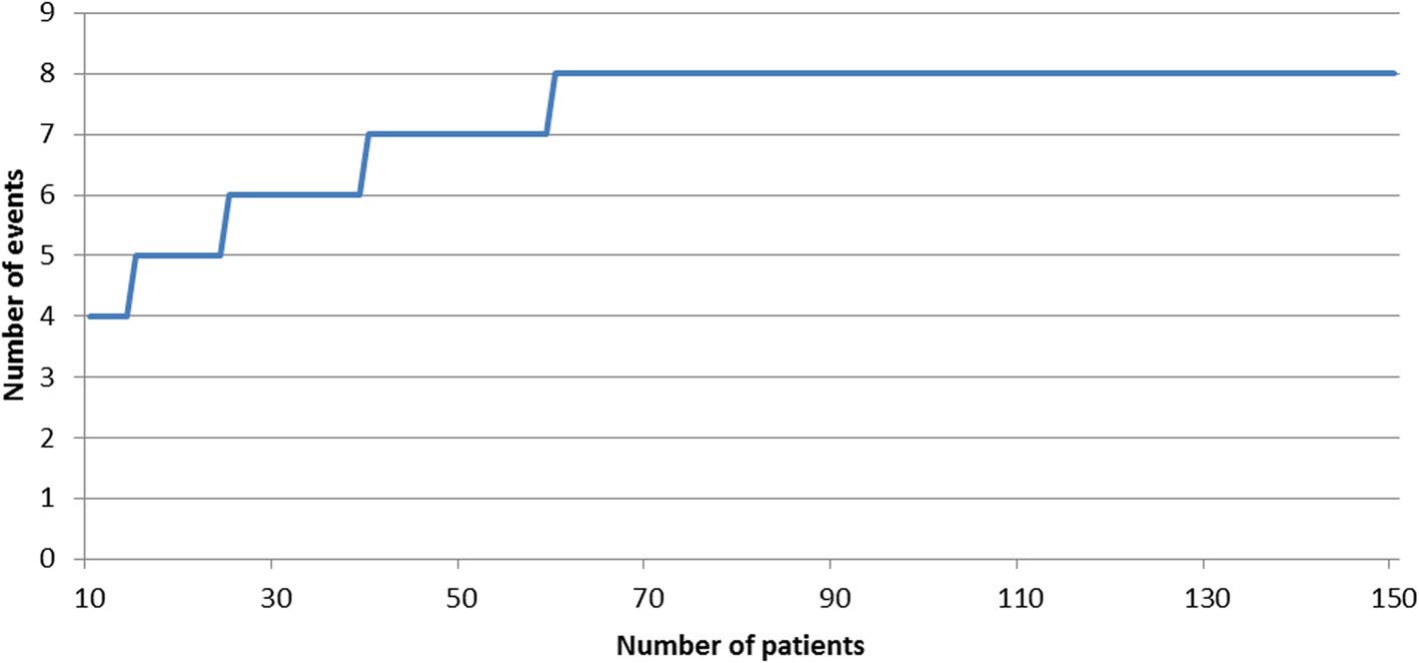
Stopping rule of SLN procedure. Legend: The stopping rule is activated when the number of groin recurrences (Y as) related to the number of patients with a negative SLN exceeds the line. * see text for all in- and exclusion criteria, **inclusion and exclusion criteria applicable to local tumour, but strong suspicion or evidence of tumour positive lymph nodes (clinical, radiological or proven with fine needle aspiration or biopsy)

In women with a first recurrence treated with a conventional IFL, the expected groin recurrence rate will be about 3%. Given the fact that the SLN procedure has many advantages over IFL, we will accept an additional failure rate of 5% for the SLN procedure in local recurrent vulvar SCC. The predictive probability design for phase II cancer clinical trials, as described by Lee and Liu, was used to develop the stopping rule for this study. Using the accompanying software (https://biostatistics.mdanderson.org/SoftwareDownload/SingleSoftware/Index/84) we calculated the minimum number of patients needed to obtain sufficient power in relation to an alpha of 0.10 and a beta of 0.20. The software calculated that we will need 150 patients with a first vulvar cancer recurrence undergoing an SLN procedure and a negative SLN. This implies that approximately a total of 240–250 women must be recruited to the study, since the SLN procedure will be unsuccessful in around 25–30% of eligible patients and the SLN will be tumour-positive in approximately 8–10% of the SLN removed (Fig. 2). Since proper data are not available calculations are based on several estimated guesses. In the course of time, it will become clear whether these assumptions are correct. A stopping rule will be implemented to ensure that when the risk failure reaches 8% the study is paused or stopped (Fig. 3).

### Statistical analysis

The primary outcome measure, groin recurrence after SLN procedure, will be calculated as the percentage of patients with a groin recurrence in the group that underwent a successful SLN procedure with a negative tumour outcome. Feasibility, the success rate of SLN detection and retrieval will be calculated in relation to the number of patients and the number of groins in which the proposed SLN surgery is successfully performed. Continuous secondary outcomes (i.e. duration of surgery, duration of hospital stay, blood loss) will be calculated using t-tests and discrete variables (complication rate) will be analysed using chi-square tests with continuity correction. All outcomes will be analysed using regression techniques. A *p*-value < 0.05 will be considered significant.

Interim analysis is not applicable and activation of the stopping rule can occur at any moment throughout the study period. (Fig. 3)

### Ethics and dissemination

The study has been approved by the Medical Ethical Committee of Erasmus Medical Centre Rotterdam and will be performed according to the standards outlined in the Declaration of Helsinki. Patients will receive verbal and written information from their gynaecologist at the moment of diagnosis. Subjects can leave the study at any time for whichever reason if they wish to do so, without any consequences. The investigator can decide to withdraw a subject from the study for urgent medical reasons. At this moment, there are no specific criteria for withdrawal. A stopping rule has been installed to ensure patient safety (Fig. 3). Study results will be offered for publication in international medical journals and on the website of the patient association for women with gynaecological cancer.

## Discussion

This study was designed to investigate the safety and feasibility of the SLN procedure in first recurrent V-SCC and has been approved by the Dutch patient association for women with gynaecological cancer in The Netherlands (Olijf). If the SLN procedure proves safe and feasible in this patient group it will contribute greatly to reducing the short and long-term side effects of vulvar cancer treatment and will have less impact on quality of life compared to the current standard treatment. In addition, we expect the study to provide a better understanding of the efficacy, side effects and pathology of recurrent vulvar cancer, and we anticipate that the observational arm of the study will generate new research questions.

## Data Availability

Research data at Erasmus MC is generated, stored and made accessible in accordance with legal, academic and ethical requirements. This study, and all persons involved, have knowledge of and comply with the most recent version of the Erasmus MC Research Code, which complies with all current laws and regulations. Data will be handled practicing the FAIR principles (Findable, Accessible, Interoperable and Reusable) according to the Handbook for Adequate Natural Data Stewardship (HANDS) developed by the Federation of Dutch UMCs. All research data will be handled confidentially in accordance with legislation and conditions imposed by The Dutch Data Protection Authority. The research data from this study will be stored in a long-term archive on secured network servers, with regular back up and limited access. In accordance with the Netherlands Code of Conduct for Scientific Practice, raw data will be stored for a period of at least 10 years. Permission for third persons to access the data will only be granted by the PI on certain conditions.

## Abbreviations

V-SCC: Vulvar squamous cell carcinoma
SLN: Sentinel lymph node
IFL: Inguinofemoral lymphadenectomy
